# Genome-wide characterization of RNA editing highlights roles of high editing events of glutamatergic synapse during mouse retinal development

**DOI:** 10.1016/j.csbj.2022.05.029

**Published:** 2022-05-18

**Authors:** Chenghao Li, Xinrui Shi, Jiaying Yang, Ke Li, Lijun Dai, Yan Zhang, Meng Zhou, Jianzhong Su

**Affiliations:** a School of Biomedical Engineering, School of Ophthalmology and Optometry and Eye Hospital, Wenzhou Medical University, Wenzhou, Zhejiang 325027, China; b Oujiang Laboratory (Zhejiang Lab for Regenerative Medicine, Vision and Brain Health), Wenzhou, Zhejiang 325001, China; c Wenzhou Institute, University of Chinese Academy of Sciences, Wenzhou, Zhejiang 325011, China

**Keywords:** RNA editing, Retinal development, Non-synonymous, Bipolar cells, Retinal ganglion cells

## Abstract

Adenosine-to-inosine (A-to-I) RNA editing leads to functional change of neurotransmitter receptor which is essential for neurotransmission and normal neuronal development. As a highly accessible part of central nervous system, retina has been extensively studied, however, it remains largely unknown how RNA editing regulates its development. Here, a genome-wide screening of high-confidence RNA editing events were performed to decipher the dynamic transcriptome regulation by RNA editing during mouse retinal development. 2000 high-confidence editing sites across eight developmental stages of retina were called. Three unique patterns (RNA-editing^high^ pattern, RNA-editing^medium^ pattern and RNA-editing^low^ pattern) were identified by clustering these editing sites based on their editing level during retinal development. Editing events from RNA-editing^high^ pattern were significantly associated with glutamate receptors and regulated synaptic transmission. Interestingly, most non-synonymous high-editing sites were mapped to ion channel genes of glutamatergic synapse which were associated with neurotransmission by controlling ion channel permeability and affecting exocytosis. Meanwhile, these non-synonymous editing sites were evolutionarily conserved and exhibited a consistently increasing editing levels between mouse and human retinal development. Single-cell RNA-seq data analysis revealed that RNA editing events prefer to occur in two main cell types including bipolar and amacrine cells. Genes with non-synonymous high-editing sites were enriched in both bipolar cells and retina ganglion cells, which may mediate retina ganglion cell differentiation by altering channel ion permeability. Together, our results provide novel insights into mechanism of post-transcriptional regulation during retinal development and help to develop novel RNA editing-guided therapeutic strategies for retinal disorders.

## Graphical abstract

**Figure:**
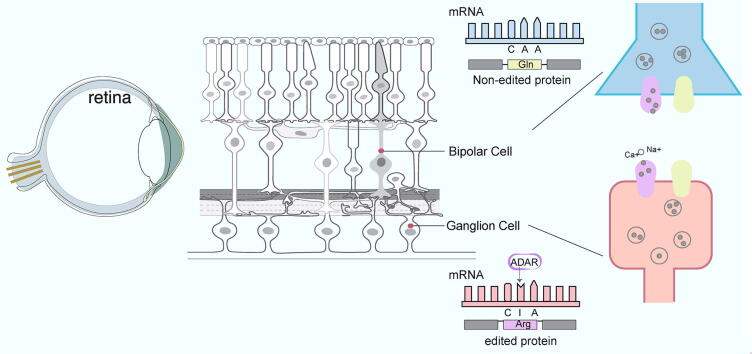

## 1 Introduction

Adenosines(A) to inosines (I) RNA editing is the most abundant form of RNA post-transcriptional modification catalyzed by ADAR (adenosine deaminase acting on RNA) enzymes which convert A to I in double-stranded RNA (dsRNA). Due to structural similarities, inosines (I) are read by the cellular machinery as guanosine (G) [1]. Editing of the RNA sequence provides an additional layer of regulation to the transcriptome, diversifying genetic information without altering genomic information. RNA editing is implicated in many aspects of RNA biology, such as splicing, stability and translational regulation [2], [3], [4].

Transcriptional profiling of genome-wide RNA sequencing confirmed a large number of RNA editing sites in many important neuronally expressed genes [5]. RNA editing sites causing non-synonymous change are frequently located in genes involved in encoding ion channels and neurotransmitter receptors, whichabundantly expressed in the brain [6]. For instance, subunits of ionotropic glutamate receptors (GluRs) where A-to-I editing creating multiple isoforms of proteins are essential for neuronal excitability [7]. Furthermore, RNA editing influences the splice process and miRNA targeting efficiency, consequently regulating neuronal gene expression [8]. In addition, editing levels increase at most editing sites during neuronal differentiation and brain maturation in a spatiotemporal manner [9]. These editing-induced regulation in the transcriptome implies that A-to-I editing is critical for neurotransmission and normal neuronal development.

As the most accessible part of the central nerve system(CNS), neural retina is an excellent model system to analyze key aspects of neurogenesis [10]. Drosophila with dADAR deletion exhibited retinal structural abnormalities with vacuolated regions. Moreover, the vacuoles become increasing in size and number during development [11]. In chick retina, the transcript for GABA type A receptor subunit α3 was found to be subjected to RNA editing, and the editing level increased from 15% at Hamburger–Hamilton stages 6.5 (st6.5) to 90% at Hamburger–Hamilton stages 6.5 (st45) and finally 95% in the adult retina, suggesting that RNA editing may play important roles in regulation of retina development [12]. Several studies reported that altered RNA editing levels may cause retina-related disorders. Glaucoma is a visual disorder characterized by progressive loss of retinal ganglion cells (RGCs), which often associated with characteristic axon degeneration in the optic nerve [13]. In adult mice, chronically elevated intraocular pressure (IOP) inhibits ADAR2 expression, leading to loss of GluA2 RNA editing that might potentially promote RGCs neuronal death in glaucoma [14].

As the most accessible part of CNS, the mechanisms underlying retinal development are highly conserved across vertebrates, making it an excellent model system for studying nerves. Although RNA editing is essential for the functions of nervous system, their dynamic changes and specific roles in retinal development and mechanisms are unclear.

In this study, we developed a framework to comprehensively characterize dynamic RNA editing events during mouse retinal development. Three distinct patterns including RNA-editing^high^ pattern, RNA-editing^medium^ pattern and RNA-editing^low^ pattern were identified. Among them, we found that RNA-editing^high^ pattern showed a clearly increasing trend during retinal development. The associated genes are significantly enriched in glutamatergic genes, which play a crucial role in neurotransmission by controlling ion channel permeability and affecting exocytosis. Moreover, non-synonymous RNA editing sites in glutamate ion receptors are associated with bipolar cells and retinal ganglion cells. All in all, our study provided a genome-wide map of RNA editing events and characterized different RNA-editing-mediated post-transcriptional modification patterns during retinal development. The novel high-confidence retina-specific editing sites expanded the known RNA editing resource.

## 2 Materials & methods

### 2.1 Data acquisition

The bulk RNA-seq data for mouse retina development were downloaded from the NCBI Sequence Read Archive (https://www.ncbi.nlm.nih.gov/sra, accession number SRP090043) [15], where high-throughput sequencing on the transcriptome of eight key developmental stages of mouse retina (E14.5, E17.5, P0, P3, P7, P10, P14 and P21) were performed.

### 2.2 Genome-wide screening of A to I RNA editing sites

We used a pipeline adapted from a previously reported method [16] to identify A to I RNA editing events. For each sample, we used BWA to map the pre-processed RNA-seq reads to the reference mouse genome (mm10) and the exonic sequences surrounding known splicing junctions from UCSC, RefSeq, Ensembl and GENCODE gene models. To remove duplicates of polymerase chain reaction (PCR), we used SAMtools to remove clonal mapped reads [17]. A to I RNA editing sites were determined as A-to-G mismatches between the RNA-seq reads and the mouse reference genome (mm10). We used SAMtools to process and pile up the mapped reads to quantify the number of editing reads. In order to obtain the high-confidence editing sites, we filtered the editing sites by the following thresholds: (1) the reads that support the A-to-G mismatches should satisfy a base quality score ≥ 25 and the mapping quality score ≥ 20; (2) the minimum read coverage should be 15; (3) mismatch rate(A-to-G mismatched reads/mapped reads) ≥ 0.02; (4) remove the intersection of mismatches and the known SNPs from dbSNP database and 1000 Genomes Project; (5) the high-confidence sites should occur at more than 50% samples.

### 2.3 The annotation of A to I editing sites

To further analyze the function of RNA editing sites, we used ANNOVAR [18] to annotate every high-confidence editing sites. These editing sites in exonic regions were also annotated by ANNOVER to obtain information on synonymous and non-synonymous editing sites. The annotation of RNA editing sites that in the repeat regions were based on the RepeatMasker table (GRCm38/mm10) [19], available at the UCSC Genome Browser (https://genome.ucsc.edu/).

### 2.4 Function enrichment analysis

We performed KEGG pathway and Gene ontology (GO) enrichment analyses by the enrichGO and enrichKEGG function in the R package clusterProfiler [20] (v3.10.1; https://github.com/YuLab-SMU/clusterProfiler). We used all mouse genes as a reference gene set to compare with edited genes for functional enrichment analysis. Only the terms of "biological process" were selected for GO enrichment analysis. Enrichment with FDR was adjusted by Benjamini-Hochberg multiple comparisons. The selected KEGG pathways related to RNA editing were visualized using the Pathview package [21].

### 2.5 Motif enrichment analysis

Motif prediction was performed using MEME-ChIP in the MEME suit (Version 5.4.1, https://meme-suite.org/tools/meme) [22], by inputting the RNA sequences from 5 bp upstream to 5 bp downstream of A to I RNA editing sites. The MEME tool was used to measure the significance of the motif by the E value [23].

### 2.6 Relation between overall editing levels and ADAR family expression

To assess the association between editing writer and editing levels, the correlations between ADAR family expression and overall RNA editing level were calculated using Linear regression models. According to previous studies [24], [25], [26], the overall editing level was defined as the ratio of total reads with G to total mapped reads in samples from different retinal developmental stages. Benjamini-Hochberg correction was applied to adjust for multiple testing to calculate significance levels.

### 2.7 Identification of RNA editing patterns during retina development

Unsupervised learning with k-means analysis was performed to cluster A to I RNA editing sites using the k-means function from package "stats". We used package "fpc" to test different numbers of clusters and measuring the resulting sum of squared errors, and then we chose “elbow point number” 3 as the optimal number of clusters.

### 2.8 Single-cell RNA-seq analysis

The expression matrix was read and transformed into Seurat object, and we filtered out cells by the following criteria: greater than 101–6000 expressed genes, <200 UMIs, more than 10% of UMIs corresponded to mitochondrial genomes. After normalization by sctransform, we performed dimensionality reduction using the PCA method. Based on the elbow plot, we chose the top 11 PC for downstream clustering and UMAP embedding. To assign cell identity labels to clusters, we used the cell clustering information from the source article of the data [27].

We downloaded fastq files and corresponding barcode lists for six stages of mouse retinal development [27] from the NCBI SRA (accession number SRP158081). Then we separated the reads for each cell type to constitute new fastq file of each cell types by the cell-specific barcode. Then we treat reads of the same cell type at one stage as reads of a sample and perform the same preprocessing as for bulk-seq analysis. To increase confidence, we used the high-confidence sites that we obtained from next-generation sequencing as the background to extract reads from each cell type. For each high-confidence editing site, the RNA editing level was defined as the number of reads with mismatches divided by the number reads that mapped to the site.

We calculated the enrichment scores of the gene sets that have RNA editing sites for each individual cell within these clusters to get the cell types that enriched the gene with RNA editing by the Addmodulesocre function from package "Seurat" [28].

## 3 Result

### 3.1 Genome-wide screening of A to I RNA editing events during mouse retinal development

**Fig. 1:**
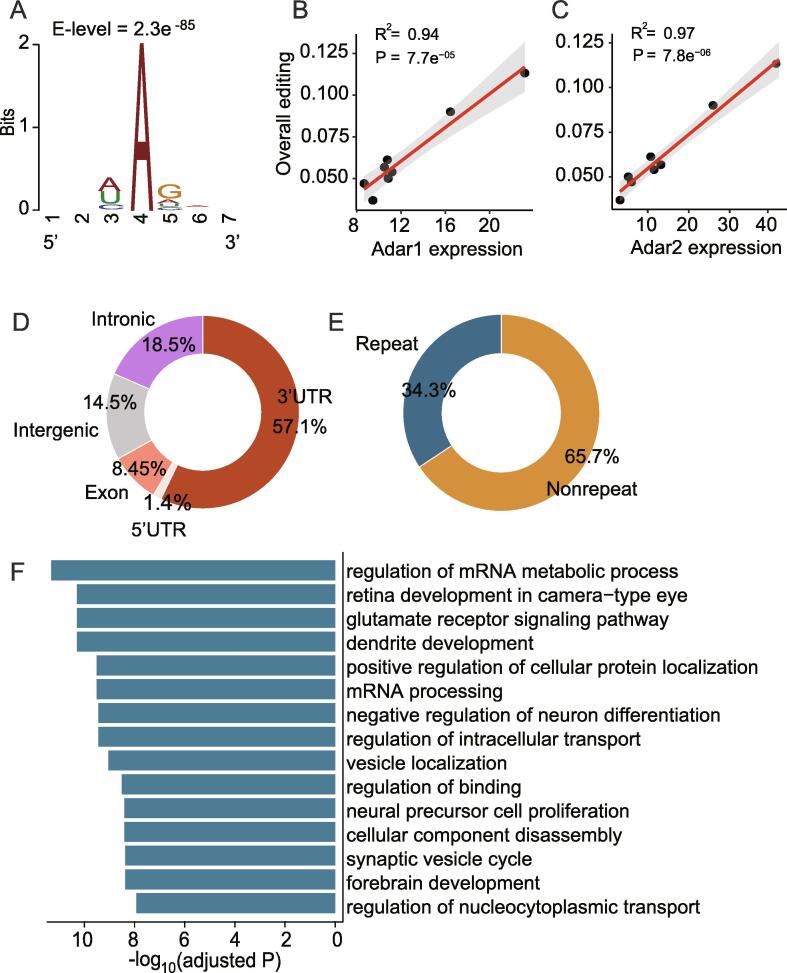
**Global analysis of A to I RNA editing sites during mouse retinal development.** A) The sequences neighboring the RNA editing sites, exhibit the pattern consistent with known Adar motif; B.C) Correlations between expression levels of Adar1 and Adar2(quantified as the number of fragments per kilobase per million mapped fragments (FPKM)) and overall editing levels; D) Distribution of A to I RNA editing sites to different genomic locations (UTR, untranslated region); E) Distribution of A to I RNA editing sites in repeat elements versus non-repeat elements; F) GO enrichment analysis of genes that have RNA editing sites.

To identify RNA editing events during retinal development, we applied our previously pipeline to de novo call A-to-I RNA editing sites across eight different developmental stages in mouse (Supplementary Fig. 1) [27]. After a series of rigorous screening (Supplementary Fig. 2), 2000 high-confidence editing sites across 8 developmental stages were detected for further analysis. Among them, 33.6% could not be found in RDAR2 database (Supplementary Fig.  3B) [28], suggesting that they were retinal specific. The most common RNA post-transcriptional modification, A-to-I RNA editing, is known to be mediated by two enzymes, ADAR1 and ADAR2, which convert adenosines to inosines. Here, we checked expression levels of the encoded genes Adar1 and Adar2 in 8 stages of retinal development (Supplementary Fig. 3C). First, consistent with brain tissues, Adar2 was highly expressed in retina (Supplementary Fig. 3C). Second, lower expression levels were observed in pre-developmental stage, while gradually increased as retina develops. Meanwhile, as expected, the overall RNA editing levels were strongly correlated with the expression of both Adar1 and Adar2 during retinal development (Fig. 1B and C).

To further verify the identified editing sites were bona fide, we examined the sequence context around the editing sites and found that they exhibited known ADAR deamination sequences with a large guanosine (G) deletion upstream of the editing site and some G enrichment downstream (Fig. 1A). Moreover, the appearance of particular nucleotides in the nearest neighbor positions of the newly discovered retinal specific RNA editing sites had same preference with the shared RNA editing site in database (Supplementary Fig. 3D). These analyses support that the editing events we called were authentic. In addition, the newly high-confidence retina-specific editing sites helped to expand the known RNA editing resource.

To examine the genome distribution of the RNA editing sites, we annotated the sites by ANOVAR tool [18] using mouse mm10 reference genome. The majority of the editing sites (57.1%) during retinal development was located in the 3′UTR (untranslated region) of the transcript (Fig. 1D). Besides, 34.3% editing sites were located in repeat region (Fig. 1E), which was coincident with prior knowledge *i.e.* in most species, a large proportion of editing events occur in repeating elements may form long double-stranded RNA structures that are more favorable for enzyme binding [29].

To explore possible functional significance of these RNA editing events across retinal development, we performed GO functional enrichment analysis. The edited genes (934 in total) containing at least one high-confidence RNA editing site were found to be significantly enriched in retinal development in the camera-type eye, dendrite development and glutamate receptor signaling pathways (p.adjust < 0.05) (Fig. 1F). Functional enrichment analysis showed that RNA editing was associated with retinal development and neural signaling. Compared with the original list of the database, the genes, including newly retina-specific editing events, were more enriched in retina development in the camera-type eye, highlighting the retina-specific properties of RNA editing which were distinguishable from brain tissues (Supplementary Fig. 3E).

To sum up, we identified 2000 high-confidence RNA editing sites across eight retinal development stages. More than third of them were retina-specific, which may have some characteristics different from that of brain although they were all enriched in neurons.

### 3.2 RNA editing sites enriched in B1 SINE family

**Fig. 2:**
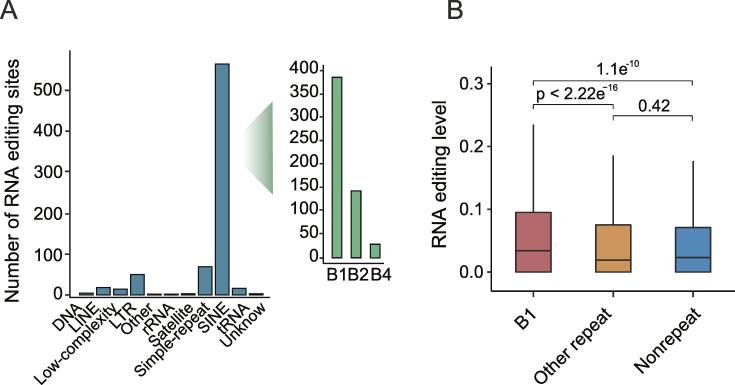
**RNA editing sites enriched in B1 SINE family.** A) Number of editing sites for each repeat family, and the green histogram shows the number of editing sites for the subfamily of SINE repeats. B) Boxplot of RNA editing level distribution among the editing sites in the B1 elements, other repeat elements and non-repeat regions. (For interpretation of the references to color in this figure legend, the reader is referred to the web version of this article.)

To further characterize these editing sites, we examined the number of high-confidence editing sites located in major repeat families in mouse. On the one hand, consistent with human beings, high-confidence RNA editing sites were primarily located in short interspersed element (SINE) (75.9%) (Fig. 2A) that were highly conserved in eukaryotic genomes[30]. Extensive evidence showed that SINE elements were transcriptionally repressed in healthy somatic cells while they were transcriptionally active during early embryonic development [31], [32]. On the other hand, long interspersed element (LINE) which is the most abundant category of repeat elements contained only few editing sites (Fig. 2A). Further analysis of SINE subfamilies (B1, B2 and B4) RNA editing sites in mouse indicated that most of them occurred at the B1 element (Fig. 2A) that originated from the 7SLRNA element in the common ancestor of primates and rodents [33].

To further dissect RNA editing events in the B1 element, editing levels between repeat family regions and non-repeat regions were compared. Result demonstrated that RNA editing level of B1 elements was significantly higher (P-value < 0.05; Wilcoxon Rank Sum Test) than other repeat elements and non-repeat regions during retinal development, while there was no editing level difference between other repeat elements and non-repeat regions (Fig. 2B).

Altogether, during mouse retinal development, high RNA editing events prefer to occur at B1 repeat elements, which may form dsRNA structure that allows ADAR enzymes to bind easily to mRNA sequences [34].

### 3.3 Identification of distinct post-transcriptional regulation patterns by RNA editing during retinal development

To explore changes of RNA editing during retinal development, we quantified genome-wide editing degree by calculating A-to-I RNA editing level across all stages. For high-confidence RNA editing sites, editing rate was defined as the ratio of reads number that supported adenosine (A) to guanosine (G) to all reads number covered the sites(G/(G + A)). We observed that the RNA editing level gradually increased during retinal development (Supplementary Fig. 4A), which was consistent with the Adar1 and Adar2 expression.

**Fig. 3:**
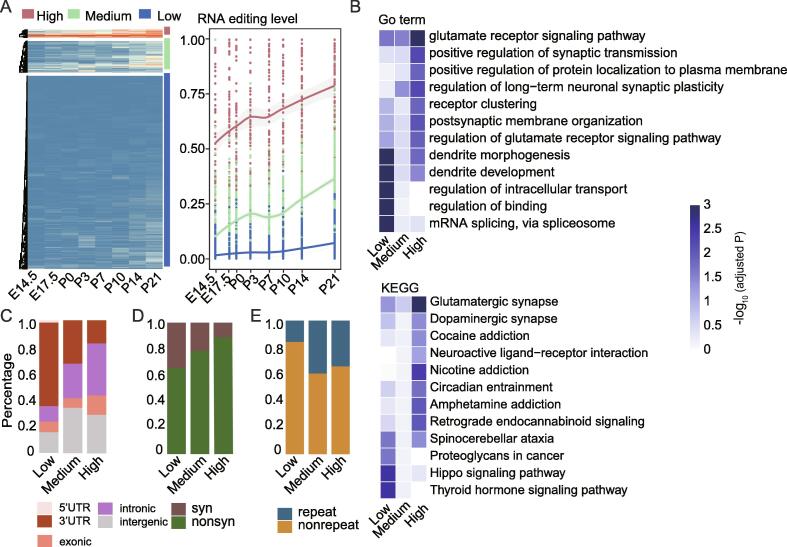
**Characteristics of different post-transcriptional regulation patterns by RNA editing in retinal development.** A) Heatmap for k-means clustering (k = 3) of 2000 high confidence editing sites.The LOESS smoothing curve represents the three editing patterns during retina development. The gray shaded areas indicate the 95% confidence interval of the smoothing curve. Red, green and blue represents RNA-editing^high^ pattern, RNA-editing^medium^ pattern and RNA-editing^low^ pattern, respectively. B) Heatmap of the GO and KEGG enrichment analysis and within the genes harboring RNA editing sites from different clusters. Color intensity indicates the adjusted P-values of enrichment tests. C) The fraction of different genomic locations in the three clusters of RNA editing sites. D) The fraction of synonymous and non-synonymous mutations in the three clusters of RNA editing sites. E) The fraction of repeat elements versus non-repeat elements in the three clusters of RNA editing sites. (For interpretation of the references to color in this figure legend, the reader is referred to the web version of this article.)

To further explore dynamic pattern of RNA editing during retinal development, K-means method was applied to cluster all high-confidence RNA editing sites based on editing level and grouped them into three co-editing clusters (red, RNA-editing^high^; green, RNA-editing^medium^; blue, RNA-editing^low^ pattern) (Fig. 3A). We chose "elbow point number" 3 (Supplementary Fig. S4 B) as the optimal number of clusters to classify editing sites with similar editing levels into one cluster. RNA-editing^high^ and RNA-editing^medium^ patterns showed a similar increasing trend during retinal development, implying their potential functional roles in neural development. Previous studies have shown that most A-to-I editing sites tend to have relatively low editing levels in the range [0, 1] [35]. Our data also indicated that most sites belonged to RNA-editing^low^ pattern and showed stable low editing levels during the whole retinal development, suggesting that these RNA editing events may play roles in basic cellular processes rather than neurodevelopmentally relevant processes.

To test our hypothesis and draw the characteristics of each RNA editing pattern, functional enrichment analysis was performed. GO enrichment analysis revealed that the RNA-editing^high^ pattern sites were highly enriched in glutamate receptor and regulated synaptic transmission. In contrast, RNA-editing^low^ pattern editing sites were significantly enriched in essential functions, such as mRNA splicing and binding-related genes (Fig. 3B). Results of KEGG pathway annotation of different editing pattern gene clusters were consistent with GO enrichment analysis.

Moreover, we found that the RNA-editing^high^ pattern are enriched in gene bodies (intronic and exonic regions) and with highest proportion of non-synonymous editing sites among all patterns during retinal development (Fig. 3C and D). And the editing-increasing patterns (RNA-editing^high^ pattern and RNA-editing^medium^ pattern) had a higher proportion of editing sites in the repeat region than the RNA-editing^low^ pattern (Fig. 3E). All in all, RNA-editing^high^ pattern, which showed rising tend during retinal development, was enriched with non-synonymous editing sites and associated with neurotransmission.

### 3.4 Non-synonymous editing events exhibit the RNA-editing^high^ pattern

To characterize non-synonymous high editing genes, we focused on glutamatergic synapse pathway, the most significantly enriched pathway of RNA-editing^high^ pattern, which was associated with excitatory synaptic transmission in central nervous system and the proliferation and differentiation of neural progenitor cells.

**Fig. 4:**
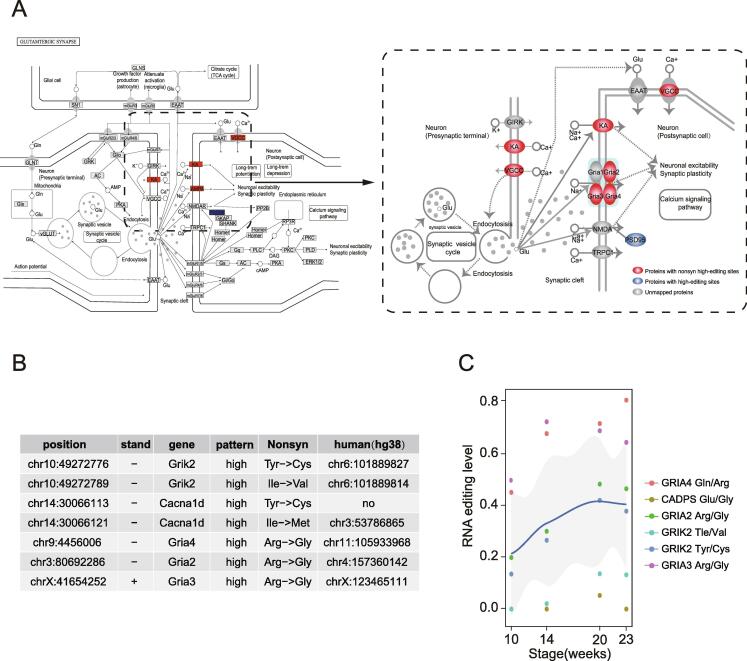
**Non-synonymous editing events exhibit RNA-editing^high^ pattern**. A) Integrated view of edited genes from glutamatergic synapse KEGG pathways (mmu04724). B) Collection of non-synonymous (Nonsyn) editing sites within ionotropic glutamate receptors and ion channel protein. Nonsyn: amino acid change; Human(hg38): non-synonymous editing sites conserved between mouse and human. C) Pattern of non-synonymous high-editing sites in human retinal development.

Notably, 71.4% genes with non-synonymous RNA editing sites were from the RNA-editing^high^ pattern and most of them (83.3%) were mapped to ion channel proteins (Cacna1d) and glutamate ionotropic receptors (Grik2, Gria2, Gria3, Gri4) (Fig. 4A). Among these genes, glutamate receptor AMPA type subunit 2 (Gria2) Q/R non-synonymous site has been reported to be involved in many neurobiological processes, in which RNA editing reduction could lead to weight loss and premature death in mouse [36], [37]. Both Grik2 Ile/Val and Tyr/Cys editing sites are located in the transmembrane domain of GluK2 (Kainate receptor subunits GluK2 encoded by GRIK2) and related to regulation of ionic permeability [38], [39]. Cacna1d mediates the entry of calcium into excite cells and participates in various calcium-dependent processes [40]. Editing at Cacna1d Ile/Met site mediated by ADAR2 enzymes resulted in a substantial reduction of Ca^2+^-feedback [41].

Interestingly, six of the eight non-synonymous editing sites were evolutionarily conserved between mouse and humans (Fig. 4B). During human retinal development, these homologous non-synonymous editing sites from RNA-editing^high^ pattern also showed increasing trend with higher editing levels compared with common sites (Fig. 4C). Moreover, we examined all 58 genes with 85 non-synonymous editing sites and found that the ion channel genes (Cadps) and glutamate receptor genes (Grik1, Grm4) were also included (Supplementary Fig. 5). These results suggested that occurrence of non-synonymous editing events on ion channel genes and glutamate ionotropic receptor genes was highly conserved across species, and exhibited RNA-editing^high^ pattern during retinal development.

### 3.5 Non-synonymous editing events were enriched in bipolar cells and retinal ganglion cells

**Fig. 5:**
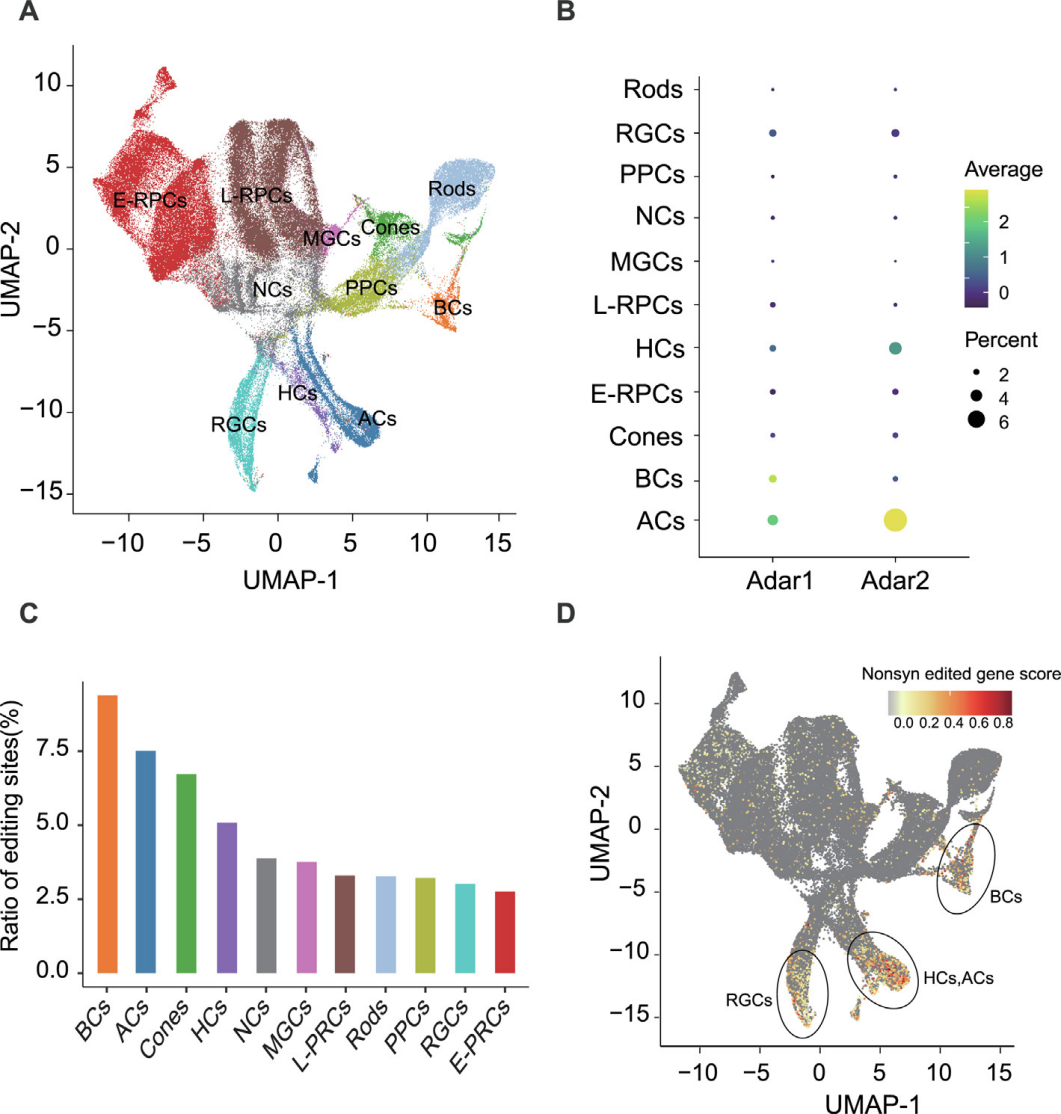
**Non-synonymous events were enriched in bipolar cells and retinal ganglion cells.** A) UMAP plot representing clusters of major retinal cell types. B) Dotplot showing the editing enzyme (Adar1 and Adar2) expression of major retinal cell types. C) The ratio of editing sites identified in various cell types. D) UMAP plot shows the non-synonymous edited genes enrichment sore in retinal cells. BCs, Bipolar cells; RGCs, Retinal ganglion cells; HCs, Horizontal cells; ACs, Amacrine cells; PPCs, Photoreceptor precursors cells; E-RPCs, Early retinal precursor cells; L-RPCs, Late retinal precursor cells; NCs, Neurogenic cells; MGs, Müller glia cells.

The major cell types of retina has been well-characterized and showed a stereotyped birth order during development [27]. To explore cell specificity of RNA editing in retina, single-cell RNA sequencing data was used for cell clustering and annotated major retinal cell types (Fig. 5A). Higher expression levels of RNA editing enzymes (Adar1 and Adar2) were observed in amacrine cells and bipolar cells compared to other cell types (Fig. 5B), demonstrating their cell-specific expression in retina.

To further explore the contribution of each retinal cell type to RNA editing events during retinal development, gene expression and RNA editing profiles of different retinal major cell types were analyzed. The enrichment scores of edited genes were calculated to in bulk-seq for each individual cell using the Addmodulesocre function of Seurat package. As indicated by the boxplot, amacrine cells possessed highest proportion of edited genes (Supplementary Fig. 6A and B). Each cell type was considered as one sample for RNA editing site identification (see Materials and Method for details), and ratio of RNA editing was calculated for each retinal cell types. Consistent with edited gene enrichment analysis and the ratios of editing sites, highest proportion of RNA editing sites was detected in amacrine cells and bipolar cells (Fig. 5C). This study demonstrated that editing enzymes are highly correlated with overall editing (Fig. 1B and C), and the higher expression of editing enzymes indicated that RNA editing events are actively occurring in amacrine and bipolar cells. Therefore, amacrine and bipolar cells were with most RNA editing events during retinal development and contributed most to overall RNA editing.

To investigate the specificity of these non-synonymous high-editing sites in various cell types, enrichment scores of these non-synonymous edited genes for each cell were calculated. We found that these non-synonymous edited genes were enriched in amacrine bipolar cells, retinal ganglion cells, HC cells and amacrine cells (Fig. 5D), in which bipolar cells and retinal ganglion cells were with highest scores (Supplementary Fig. 6C). Previous studies showed that during excitatory synaptic transmission, the bipolar cells expressed ionotropic glutamate receptors to deliver glutamate to retinal ganglion cells [42], [43], [44]. In addition, glutamatergic transmission between bipolar cells and retinal ganglion cells (RGCs) could regulate the development of RGCs dendrites to the ON and OFF sublayers of the IPL [45]. Non-synonymous RNA editing events of glutamate ionotropic receptor result in alteration of ion permeability of glutamate ion receptors [39]. These results suggested that the RNA editing in retinal ganglion cells may play important roles in developmental stratification. For example, the Gria2 Q/R non-synonymous editing site that identified here (Fig. 4B) has been reported to regulate the differentiation of human neural progenitor cells (NPCs) by affecting the calcium-permeability of glutamate ion receptors [46]. Together, our results indicated that non-synonymous RNA editing sites in glutamate ion receptors were associated with bipolar cells and retinal ganglion cells. And these non-synonymous RNA editing events may regulate signaling and cell differentiation by altering channel ion permeability.

## 4 Discussion

To our best knowledge, this is the first study with comprehensive characterization of A-to-I RNA editing events in mouse retina. In addition to identification of novel high confidence retina-specific editing sites, we featured distinct dynamic post-transcriptional regulation patterns during retinal development. Edited genes from RNA-editing^high^ pattern were significantly enriched in glutamatergic genes and ion channel genes, which is consistent with previous findings that high editing sites were mainly detected in neurons [47], [48], [49]. RNA editing sites from RNA-editing^low^ patterns were observed in genes involved in RNA binding and splicing. As reported, RNA binding proteins such as FMRP and ILF3 could directly regulate RNA editing by affecting the interaction between ADAR enzyme and RNA sequence [49]. What's more, RNA editing events could both stimulate or inhibit splicing efficiency by altering *cis*-acting signals that regulate splicing activity and destabilize double-stranded structures [50].

In humans, most RNA editing events were found in transcribed repeat elements [51]. Among all the repeat types, Alu repeat element can form stable dsRNA structures, making it the major substrates for editing enzymes. Our results confirm mouse B1 repeat element that the sequence homology element with human Alu repeats contribute significantly to RNA editing events with clustered distribution and higher editing levels [52]. Although Alu and B1 derived from same origin, they differ in length and number, making it more difficult for mice to form long dsRNA to affect ADAR enzyme binding and resulting in differences in the number of editing sites between mouse and human [53]. Previous studies have shown that expression of SINE repeat element increased during neural activity and embryonic development [31], [54], but the effect of RNA editing on SINE in these two processes needs further investigation.

Non-synonymous mutations were largely considered deleterious because they resulted in the change of amino acid sequence. However, these non-synonymous editing sites, from RNA-editing^high^ patterns in retinal development, play a crucial role in neurotransmission by controlling ion channel permeability and affecting exocytosis [55], [56], [57], [58]. These non-synonymous editing sites showed high editing levels relative to other editing sites during retinal development, and some of them even reached to 100% editing in adults (such as Gria2 Q/R site) [57]. Notably, these editing sites were conserved across species, suggesting a selective advantage because newly acquired deleterious sites would be actively eliminated while beneficial sites would be retained in evolution.

Comparative analysis of vertebrate and invertebrate genomes suggests that a substantial proportion of vertebrate genes had no homologous genes in the invertebrate genome [59]. The homologous non-synonymous editing sites shared between mouse and drosophila may play a fundamental role. Differences in the homologous non-synonymous editing sites among mouse, human and drosophila also indicated sites gain and loss during evolution. Previous studies showed that octopuses adapted to different temperature extremes by affecting the gating kinetics of potassium ion channel through different editing levels of non-synonymous editing sites in potassium channels [60]. These results suggested that non-synonymous editing is associated with environmental adaptation and evolutionary benefits, and the specific mechanisms require further study. To adapt to light and dark environments, eyes have to work over a wide range of light levels. It was reported that A-to-I RNA editing events in the Suprachiasmatic nucleus (SCN) regulated the phase response of mouse circadian system upon light induction [61]. Therefore, it is worth to explore the regulation of RNA editing in retinal adaptation to different light environments in the future studies.

During our investigation, we suffered from short reads, low coverage and easy mismatches when using single-cell data for RNA editing analysis. To address these issues, we merged reads from the same cell types to increase coverage and used RNA editing profiles from bulk-seq as background to avoid false sites generated by mismatches. With the development of single-cell sequencing technology, we may solve these problems possibly at the sequencing level and identify more cell-specific editing sites. Single-cell analysis of RNA editing found that non-synonymous editing was associated with bipolar cells and retinal ganglion cells. Consistent with previous reports, our results revealed that non-synonymous RNA editing could modulate the electrical signaling pathway between bipolar cells and retinal ganglion cells by affecting glutamate ion receptor permeability during stimulus transmission.

## Declaration of Competing Interest

The authors declare that they have no known competing financial interests or personal relationships that could have appeared to influence the work reported in this paper.
