# Supplementary material for: Genome-wide characterization of RNA editing highlights roles of high editing events of glutamatergic synapse during mouse retinal development
Source: Comput Struct Biotechnol J. 2022 May 18;20:2648–56. doi: 10.1016/j.csbj.2022.05.029 (PMC9162912; doi:10.1016/j.csbj.2022.05.029)
Supplement: Supplementary data 2 [file mmc2.pdf]

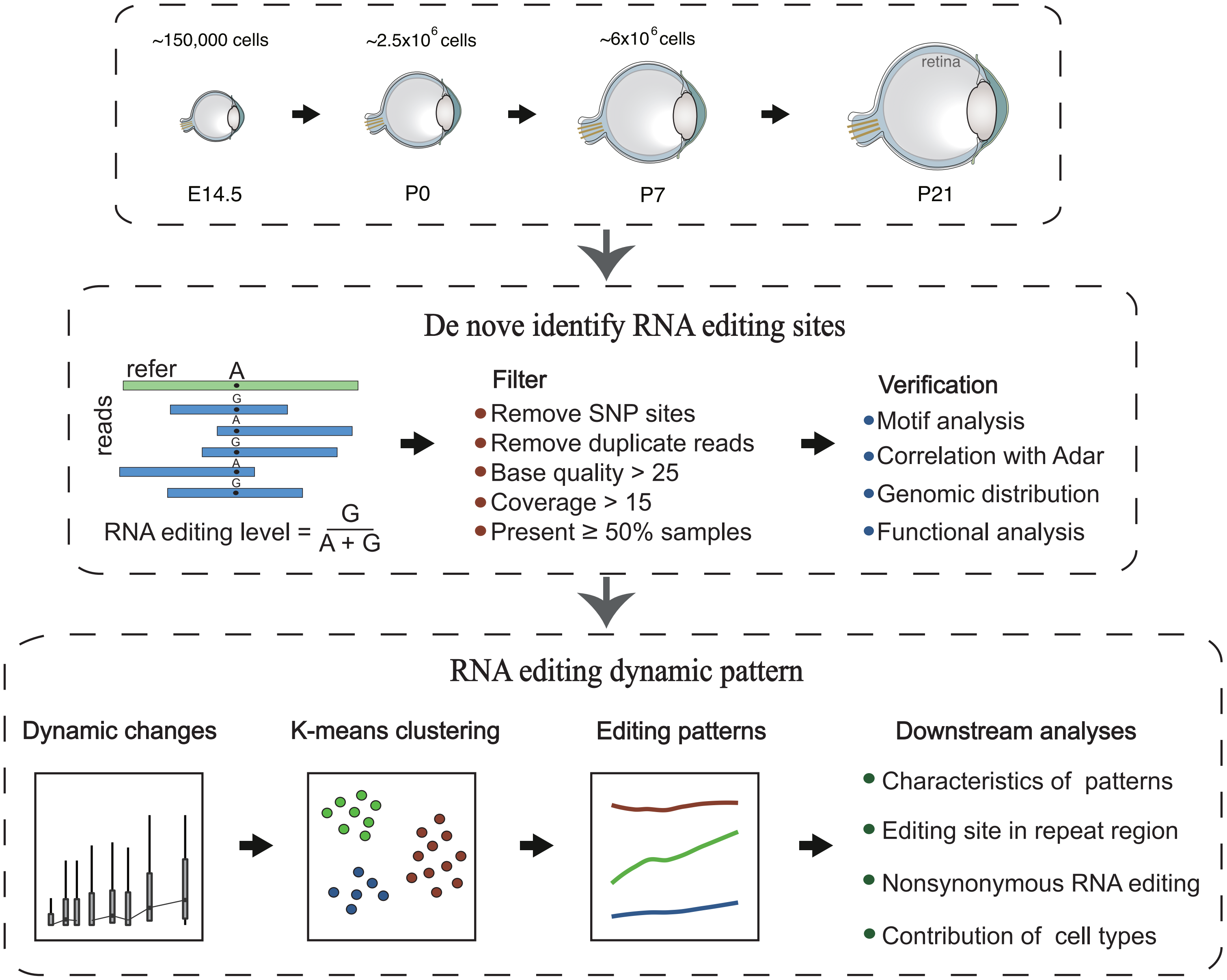

~150,000 cells
~2.5x10^6 cells
~6x10^6 cells
retina
E14.5
P0
P7
P21
De nove identify RNA editing sites
refer
A
reads
G
A
G
A
G
RNA editing level = G / A + G
Filter
Remove SNP sites
Remove duplicate reads
Base quality > 25
Coverage > 15
Present ≥ 50% samples
Verification
Motif analysis
Correlation with Adar
Genomic distribution
Functional analysis
RNA editing dynamic pattern
Dynamic changes
K-means clustering
Editing patterns
Downstream analyses
Characteristics of patterns
Editing site in repeat region
Nonsynonymous RNA editing
Contribution of cell types
