# Supplementary figures and images for: Genome-wide characterization of RNA editing highlights roles of high editing events of glutamatergic synapse during mouse retinal development
Source: Comput Struct Biotechnol J. 2022 May 18;20:2648–56. doi: 10.1016/j.csbj.2022.05.029 (PMC9162912; doi:10.1016/j.csbj.2022.05.029)

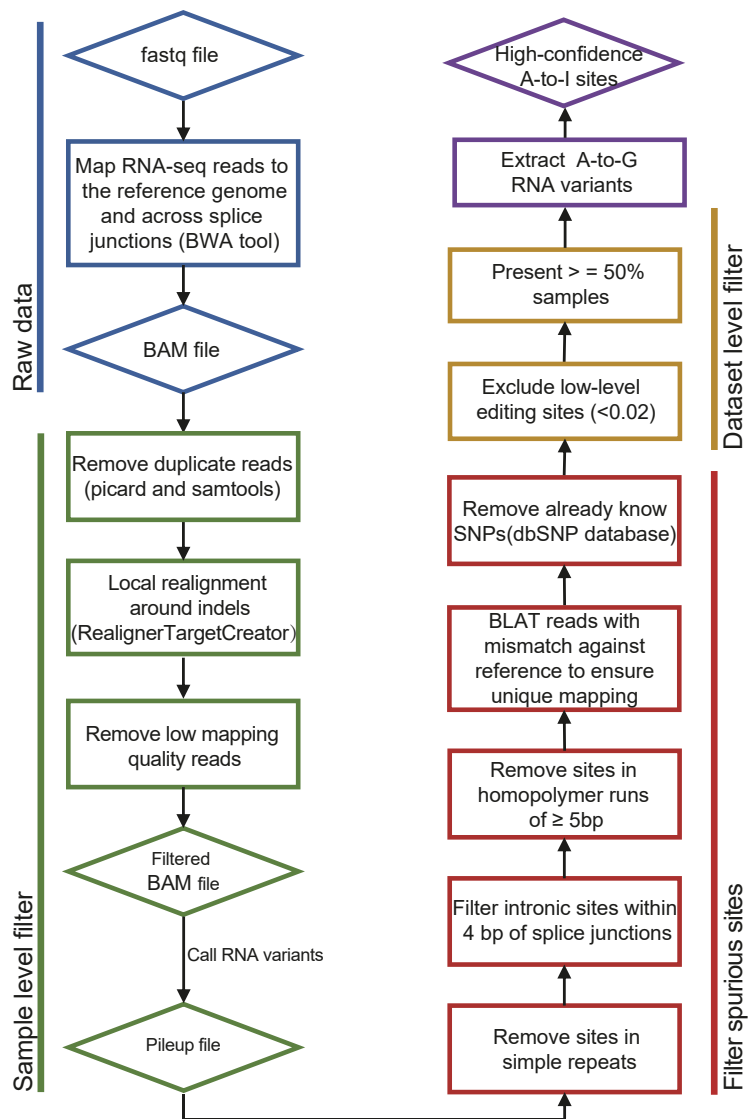

Supplement: Supplementary data 3 [file mmc3.pdf]

**A**

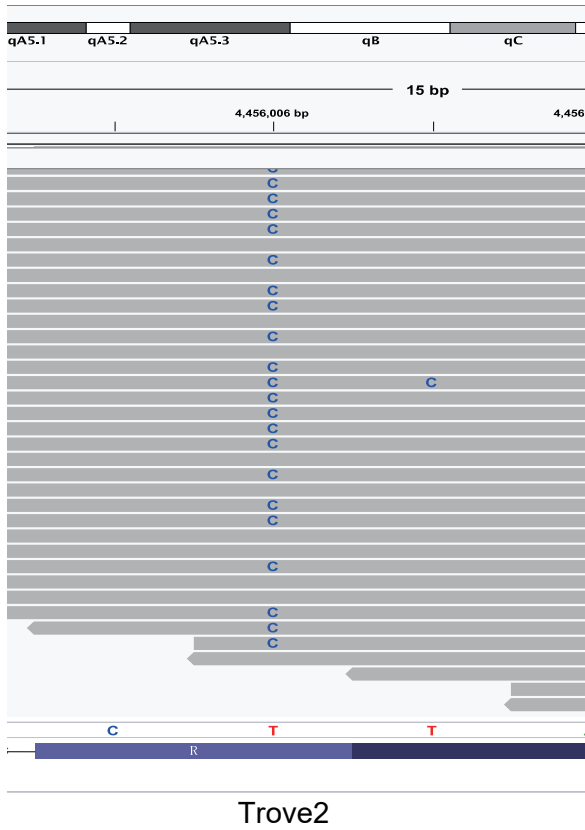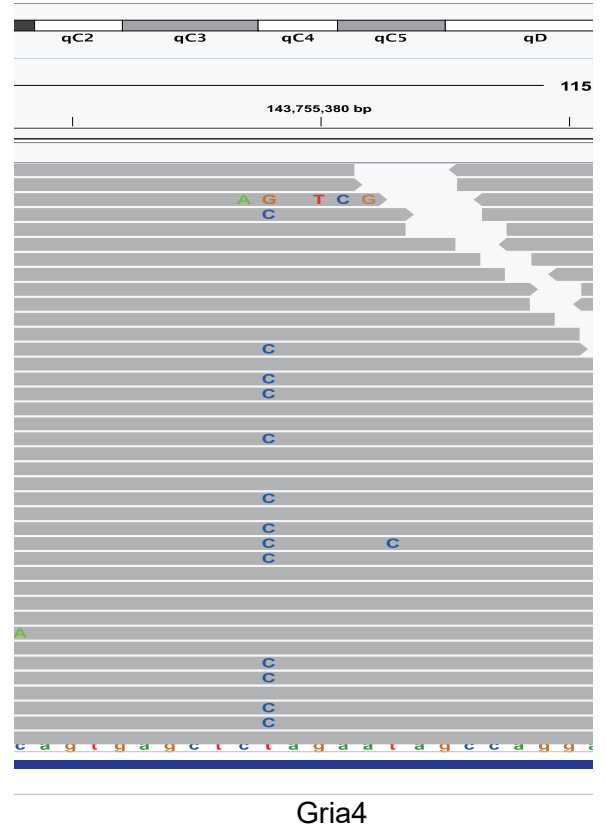

**B**

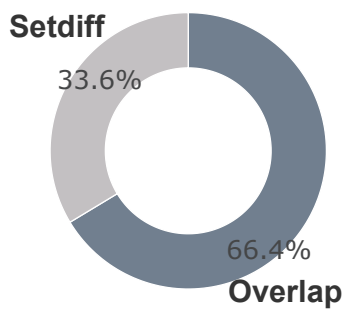

**C**

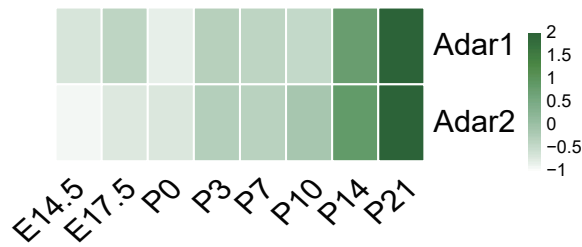

**D**

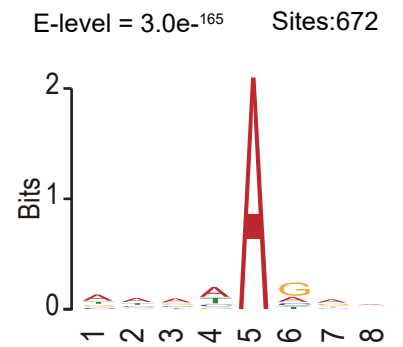

**E**

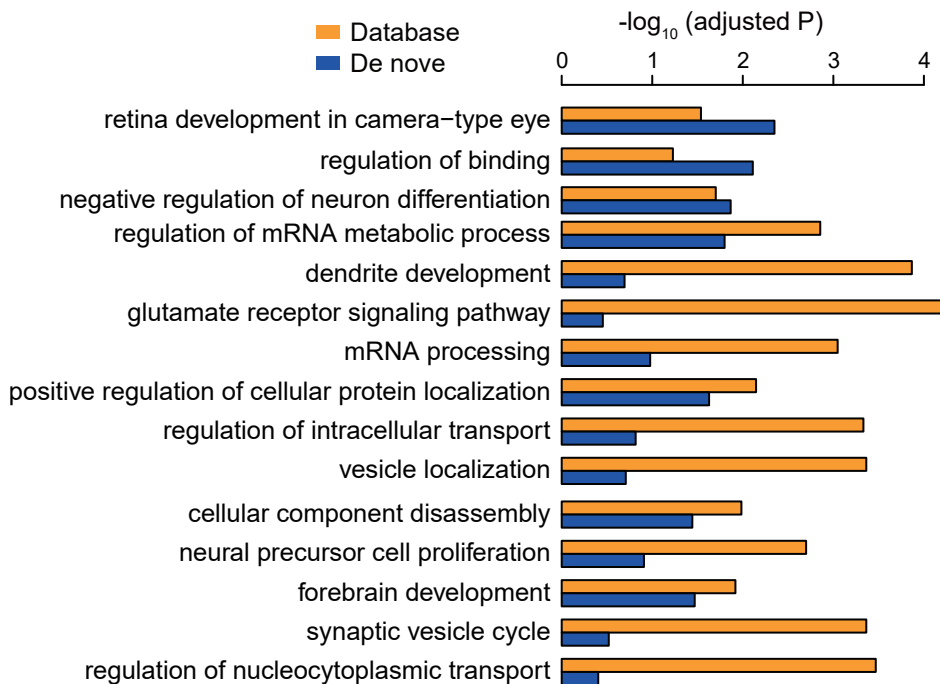

Supplement: Supplementary data 4 [file mmc4.pdf]

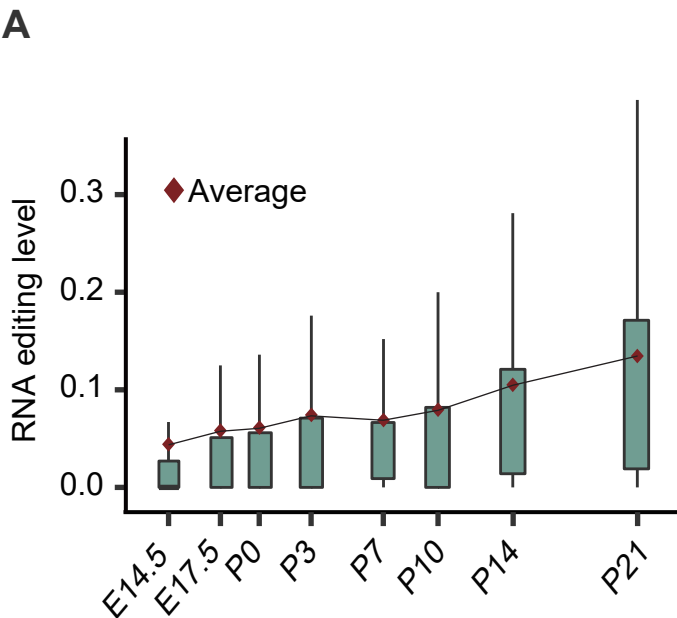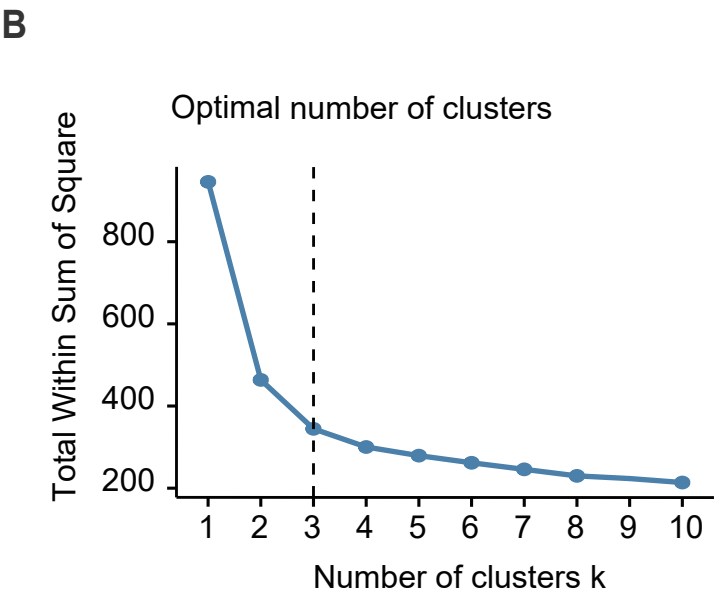

Supplement: Supplementary data 5 [file mmc5.pdf]

**A**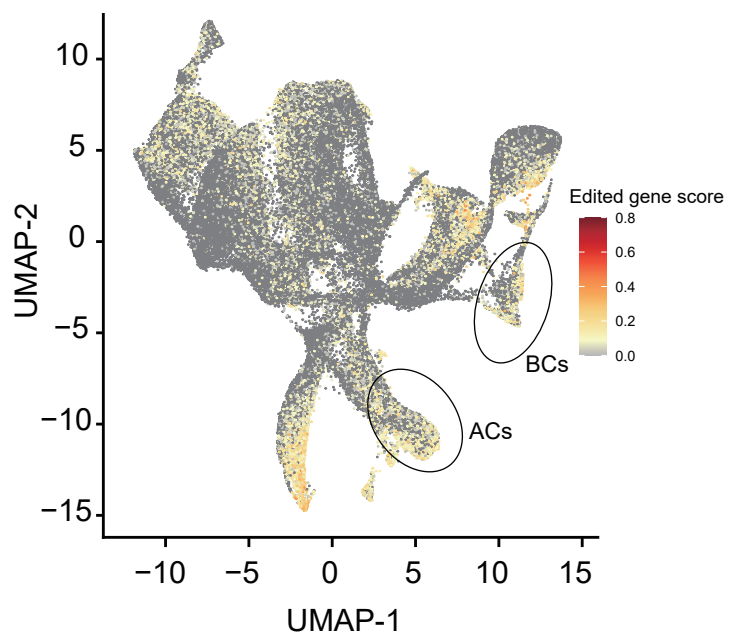**B**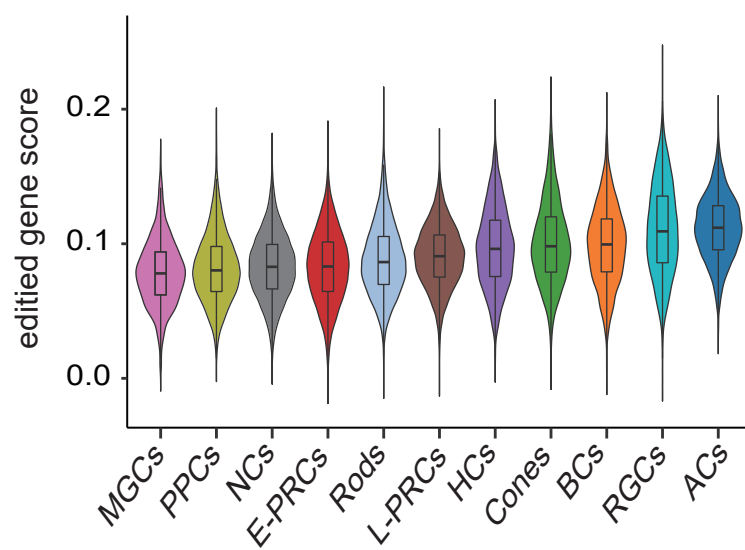**C**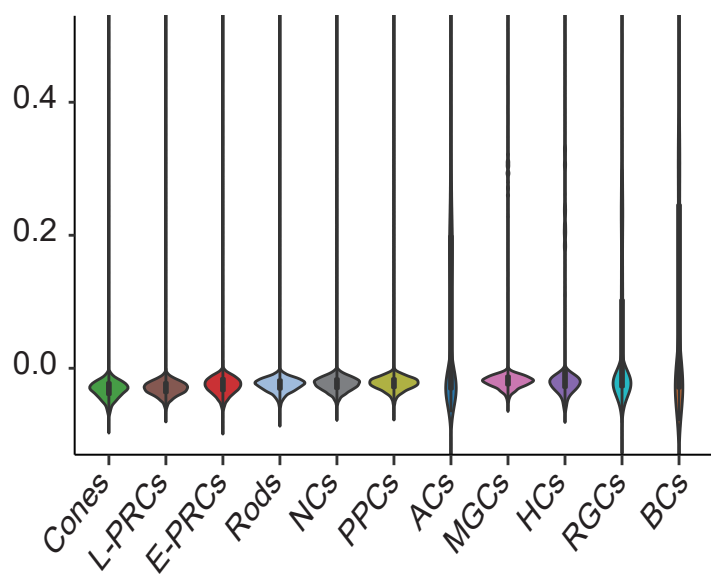

Supplement: Supplementary data 7 [file mmc7.pdf]
