# Supplementary material for: Genome-wide characterization of RNA editing highlights roles of high editing events of glutamatergic synapse during mouse retinal development
Source: Comput Struct Biotechnol J. 2022 May 18;20:2648–56. doi: 10.1016/j.csbj.2022.05.029 (PMC9162912; doi:10.1016/j.csbj.2022.05.029)
Supplement: Supplementary data 6 [file mmc6.pdf]

position	stand	gene	pattern	Nonsyn	human(hg38)
chr16:87940543	−	Grik1	medium	Gln−>Arg	no
chr14:12411582	−	Cadps	low	Glu−>Gly	chr3:62438132
chr14:12822474	−	Cadps	low	Ser−>Gly	no
chr17:27502795	−	Grm4	low	Gln−>Arg	chr6:34133126
chr10:49244334	−	Grik2	low	Met−>Val	no
